# Perspectives on the future of the profession of microbiology

**DOI:** 10.1128/msphere.00654-25

**Published:** 2025-11-25

**Authors:** Todd L. Kelson, Heidi B. Kaplan, Michelle Dziejman, Shilpa Gadwal, Irene Hulede, Jennifer Bennett, Roger Greenwell, Shelley Payne, Kyle MacLea, Eric Miller, Diana L. Vullo, Rebecca Sparks-Thissen, Astral Bertolio, Jorge Cervantes

**Affiliations:** 1 Department of Biology, Brigham Young University Idaho 124512, Rexburg, Idaho, USA; 2 Department of Microbiology and Molecular Genetics, The University of Texas Health Science Center at Houston 12340, Houston, Texas, USA; 3 Department of Microbiology and Immunology, University of Rochester Medical Center 548028, Rochester, New York, USA; 4 Department of Education, American Society for Microbiology 11003, Washington, DC, USA; 5 Department of Biology, and Earth Science, Otterbein University 638192, Westerville, Ohio, USA; 6 Undergraduate Biotechnology Program, Worcester State University 8719, Worcester, Massachusetts, USA; 7 Department of Molecular Biosciences, The University of Texas at Austin 196204, Austin, Texas, USA; 8 Department of Biological Sciences and Biotechnology, University of New Hampshire 3067, Durham, New Hampshire, USA; 9 Department of Plant and Microbial Biology, North Carolina State University 6798 , Raleigh, North Carolina, USA; 10 Universidad Nacional de General Sarmiento 28222, Los Polvorines, Argentina; 11 Indiana University School of Medicine 12250, Indianapolis, Indiana, USA; 12 American Society for Microbiology Young Ambassador to Colorado, Boulder, Colorado, USA; 13 Dr. Kiran C. Patel College of Allopathic Medicine, Nova Southeastern University 503842, Fort Lauderdale, Florida, USA; Shenzhen Institute of Synthetic Biology, Chinese Academy of Sciences, Shenzhen, China

**Keywords:** microbiology, workforce, professional development, technological advancements, interdisciplinary collaboration

## Abstract

The Profession of Microbiology (POM) embodies the bulk of the American Society for Microbiology (ASM) members and represents the career preparation arm of the ASM for academia, industry, and clinical lab professions. The ASM Council on Microbial Sciences hosted a virtual retreat in 2025 to identify the future of the POM. The retreat presentations centered on workforce development, professional development, innovations in technology, and interdisciplinary collaborations. Various aspects were identified, such as the need to prepare for careers in industry, as an important goal of future training. It was also clear that scientists, in all walks of life, need professional development training throughout their careers, from early trainees to senior scientists. Innovations in technology warrant continual training to keep abreast of global issues. Finally, the need for science advocacy and the ability to effectively communicate science to citizens is important. The ASM is best suited to leading the way in the recruitment of young scientists to the field of microbiology and providing the necessary training to keep them ahead of the changing technologies. As such, the ASM is poised to prepare its members for a quickly changing career workplace, one that will require collaboration between the many sciences and the community.

## INTRODUCTION

Ten years ago, a global survey was offered to people for whom microbiology forms a part of their profession, asking “What is a microbiologist?” (1). The results revealed the multidisciplinarity and evolving nature of the microbiology academic workforce, as well as the contribution of a range of different disciplines to the field of microbiology. During the 21st century, the microbiological workforce has been increasingly enriched by scientists trained in diverse fields, reflecting the discipline’s cross-cutting and essential role in addressing global challenges (1, 2). As microbiology continues to intersect with emerging technologies and societal needs, it stands not only as a cornerstone of scientific advancement but also as a dynamic career path.

The American Society for Microbiology (ASM) released almost 30 years ago an optimistic employment outlook for graduates with doctoral degrees in microbiology (3), in which a steady momentum was projected with an estimated 6% annual increase in job opportunities. However, a recently released ASM Workforce report (4) revealed that much of the expansion is unfolding in industry and undergraduate teaching rather than in traditional academia. The report showed that during the past 25 years, the percentage of Ph.D. microbiologists working in tenure-stream academic positions fell from 22% to 16%, as government employment increased from 7% to 12%, and a considerable number of Ph.D. microbiologists (35%–45%) were working in industry. The report also found that while there has been a 20% growth in doctoral degrees in microbiology, the pace has slowed in the past 6 years.

Microbiology is undergoing a rapid transformation, propelled by global events and technological innovation that have reshaped both research priorities and public perceptions. Advances in genomics and other molecular tools are opening unprecedented avenues for inquiry, expanding the scope and resolution of microbiological research. A central effort is the promotion of public microbial literacy, viewed as essential for informed engagement with the pressing health and environmental issues of the 21st century (5).

Along with these transformations, clinical microbiology is poised for significant advancement, driven by the convergence of cutting-edge research tools, the restructuring of clinical laboratory operations, and enhanced communication between clinicians and microbiologists. These developments promise to reshape clinical practice, fostering more integrated and responsive diagnostic approaches that reflect the complexity of microbial threats and the sophistication of contemporary science (6).

Professional societies have long played a foundational role in shaping the discipline of microbiology. Pioneers such as Robert Koch, Louis Pasteur, and Shibasaburo Kitasato transformed medicine and public health, and despite any existing rivalry, they associated and worked together (7). Since then, microbiologists have organized themselves into formal associations to foster collaboration, advance knowledge, and promote standards of practice. Among these, the ASM stands as the oldest microbiological society in the world and one of the longest-standing biological societies globally (8).

In the modern era, such societies serve not only as custodians of scientific tradition but as dynamic platforms for career and professional development. ASM’s Council on Microbial Sciences (COMS) Profession of Microbiology (POM) community exemplifies this role by supporting a diverse scientific workforce involved in microbiology (9). Topics, such as science communications, visibility tools, and strategies for securing federal funding, are complemented by networking events, research showcases, and technology expos during the annual ASM Microbe meeting through the ASM POM track (track leader at ASM Microbe 2025, Rebecca Yee). These initiatives cultivate community, spark innovation, and allow microbiologists at all career stages to engage meaningfully with the latest developments in microbial science.

The previous ASM COMS POM report (10) recommended that the society provide opportunities for sustained engagement at all career levels within ASM, develop the leadership skills of the existing society leaders, build an effective volunteer network, provide Branch leaders with strategic outreach plans, and encourage collaboration with other professional societies. It also stressed the goal of developing a robust, diverse, and growing membership into which all members at all levels can be fully engaged by accessing resources in leadership, mentoring, and career development.

Here, we describe the organization of the 2025 POM Retreat and share our findings with the community of microbiologists.

## RETREAT ORGANIZATION

### Objective of the retreat

The primary objective of the retreat was to engage leading experts, researchers, and professionals in collaborative discussions aimed at envisioning the future trajectory of the microbial sciences over the next 10 to 15 years. Participants explored emerging scientific trends, anticipated technological advancements, and identified critical challenges and opportunities likely to shape the profession. The retreat served as a strategic forum for generating innovative ideas and long-term goals to guide education, research, and workforce development in microbiology.

### Committee organization and theme selection

Representative members of ASM’s COMS and staff formed a planning committee to discuss important issues arising in the POM, and how ASM could address those concerns. This committee identified several areas of concern, and four major themes and focus questions were developed by consensus and discussion:

- Workforce development: how can an effective workforce be developed for diversifying career paths?
- Professional development: what are the unique needs for professional development at different career stages?
- Technological advancements: how can microbiology professionals be trained for rapid technological advancements?
- Interdisciplinary collaboration: how can scientists at different career stages develop interdisciplinary collaborations?

### Retreat format and speaker selection

The committee developed and organized a retreat composed of four virtual sessions over a period of 4 months (February through May 2025). For each session, three speakers with outstanding reputations and expertise in that topic were invited to give a short talk (15–20 minutes). This was followed by a question and answer period. Subsequently, small discussion groups were facilitated by one POM Committee member, who initiated a dialog using a set of prompt questions to focus the discussion. This approach helped to prime the participants and encouraged engaging discussions. At the end of each session, the main messages from each discussion group were shared with all the attendees before adjourning.

The following sections describe the speakers and topics presented during each session, along with the set of questions used in the small groups, and the demographics of participants.

## SESSION 1—WORKFORCE DEVELOPMENT: HOW CAN AN EFFECTIVE WORKFORCE BE DEVELOPED FOR DIVERSIFYING CAREER PATHS?

### Speaker highlights

Donna Ginther, Ph.D., director, Institute for Policy and Social Research, University of Kansas, spoke about “The Diversity of Career Paths for Microbiologists.” She reported that since the COVID-19 pandemic, there is a growing need for microbiologists and that more microbiologists are working in industry jobs than ever before (2). The employment trend shows a continued movement from academia to industry, but she raised the question: are we adequately preparing trainees for the industry sector? She emphasized the need to prepare mid- and senior-career scientists, in addition to early career scientists.

Roger Greenwell, Ph.D., associate professor, former coordinator of Undergraduate Biotechnology Program, Worcester State University, focused on “Workforce Development Considerations for the Future of Microbiology” by addressing how academia and industry can improve and increase their interactions. He emphasized the need for microcredential and certificate programs to increase the pipeline of new trainees. These programs help prepare trainees and early-career scientists for industry jobs by providing the training and skills needed to enter the workforce. He suggested that ASM members consider involvement in advisory boards to develop partnerships between academia and industry.

Piyush Kumar, Ph.D., instructor for environmental medicine and climate science, Icahn School of Medicine at Mount Sinai, talked about “Future Directions and Scientific Needs for Microbiology.” He focused on the need to strengthen abilities across the field in the analysis of large data sets. One approach is to encourage bioinformatics specialists to join the field, and another is to instruct more trainees in data analysis. Dr. Kumar proposed to promote the recruitment of students, as young as K-12, into careers in STEM. Finally, he urged greater support for international students to further their education in the United States.

#### Small group discussion

Five virtual breakout rooms were available, and participants could attend one of the following topics: Industry, Government Agencies, Science Policy and Regulatory, Teaching and Education, and Clinical Microbiology/Diagnostics and Lab Medicine. Each group addressed two prompt questions: how do we prepare the next generation of microbiologists for all career pathways, and what are the aspects that go into “job satisfaction?”

### Key takeaways

Careers in industry require transferable skills, including, but not limited to, storytelling, budget management, communication abilities, team building, and employee management. ASM can provide plenary sessions at its annual meeting to teach some of these transferable life skills. They can also provide plenary sessions to teach skills related more specifically to industry careers, including microbial preservation, biosafety, microbial risk, and the industry environment in general. Private industry could consider offering undergraduate student internships that are longer in length and provide training that will translate to their workplace, thereby preparing students for future careers outside of academia. Exposure to jobs in industry can be facilitated by career fairs, curriculum instruction, and partnerships between academia and industry (Table 1).

**TABLE 1:** Summary of workforce development small group discussions

Topic	Suggestions
Industry	Provide training on/highlight transferable skills needed to succeed in industry and academia Increase awareness/partner with industry on long-term industry internships Prepare for future careers by learning the skills now (because the job market is changing) Expose microbial scientists to jobs in industry via partnerships, career fairs, and integrations into the curriculum Industry-related sessions at ASM Microbe, including preservation, biosafety, microbial risk, and industry environment
Government Agencies	Define the jobs in government (Department of Defense, Food and Drug Administration, CDC, and others) and make future applicants aware of what these jobs are and how to prepare for them Foster internships and partnerships with academia
Science Policy and Regulatory	Address the growing need of advocacy in the (local, state, and national) government arena
Teaching and Education	Collaboration with non-microbiologists begins with teaching them the vocabulary of the field There will always be jobs in academia, so speak optimistically about these jobs to future applicants
Clinical Microbiology/Diagnostics and Lab Medicine	Stay up to date with medical technology

Careers in government labs include the Department of Defense, the Food and Drug Administration, and the Centers for Disease Control and Prevention. Students are not always aware of these career positions. Advertising these careers at the undergraduate and graduate training levels can help students prepare the skills needed to be competitive. This could be accomplished by encouraging students to attend webinars and by providing information on how to apply.

Careers in science policy and regulation should be better communicated because of the need for science advocacy at all levels of government (local, state, and federal). This can be accomplished by communicating this need for advocacy and by training students in these careers.

The discussion on teaching and education positions highlighted the need to appreciate interdisciplinary values and emphasize collaboration with others who have limited knowledge of microbiology to enhance overall success. Many good jobs are available in academia, and it is important to optimistically encourage those interested, as there will be a continual need for educators.

Careers in clinical microbiology, diagnostics, and laboratory medicine will require that we stay current with new technologies (i.e., DNA sequence analysis) in order to provide the most up-to-date resources to diagnose and treat infectious diseases.

## SESSION 2—PROFESSIONAL DEVELOPMENT: WHAT ARE THE UNIQUE NEEDS FOR PROFESSIONAL DEVELOPMENT AT DIFFERENT CAREER STAGES?

### Speaker highlights

Amy Vollmer, Ph.D., biology professor emeritus, Swarthmore College, spoke about “Mentoring and Networking: Two Professional Lifelines for All Stages of Your Career.” Dr. Vollmer emphasized the importance of developing networks throughout your career and seeking advice from people whom you admire. She explained that mentors are highly respected and sought after, so their time might be limited.

Brian Murphy, Ph.D., professor, College of Pharmacy, Pharmaceutical Sciences, University of Illinois, Chicago, talked about “Do Universities Serve their Communities?,” which addressed this question with an emphasis on community outreach. He described his lab’s partnerships with local community centers, such as the Boys & Girls Club of Chicago, to perform high-end biomedical research, which could serve as templates for others. Everyone involved in the program found it very rewarding. Dr. Murphy stressed the value of volunteering with K-12 students because retention can be increased when they are introduced to STEM careers at a young age.

Brooke Bissinger, Ph.D., product manager, Seed Treatment Biologicals & Inoculants, BASF, spoke on “Mid-Career Professional Development in Industry and Beyond.” She represented the industrial workplace and discussed professional development in industry. She described the advantages of incremental growth, which is making a few changes at a time, in contrast to transformational growth, in which you jump into the deep end and make huge changes all at once. She concluded by encouraging everyone to devote time to improving transferable skills that are useful in all workplaces, including collaboration, time management, creativity, communication, and emotional intelligence.

#### Small group discussion

Four virtual breakout rooms were available: Trainee, Early-Career Scientist, Mid-Career Scientist, Senior-Career Scientist/Emeritus. Each group addressed three prompt questions: what challenges do professionals face in their careers, what kinds of professional development (both technical and non-technical) are needed to address those challenges, and what role can ASM play in addressing these challenges?

### Key takeaways

Trainees were defined as undergraduate students, graduate students, and postdoctoral fellows. Exposure to various career opportunities should be offered early so as to give ample time to prepare. This might include course-based undergraduate research experience (CURE) labs at the undergraduate level, career fairs, more opportunities for internships, and skill training workshops. ASM can offer short courses, webinars, and online training addressing the need for early preparation. Since many undergraduate students attend ASM regional meetings more often than the national meeting, efforts can be targeted to the Branches to find trainees early in their careers (Table 2).

**TABLE 2:** Summary of professional development small group discussions

Topic	Suggestions
Trainees	Outreach to STEM students in high school and college to make them aware of what jobs there are and how to prepare for them Retention of STEM students via CUREs Access to internship opportunities Identify and teach transferable skills via short courses, webinars, and online training, as well as partnering with Regional ASM Branches
Early Career Scientists	Find and utilize mentors effectively Guidance to prepare for tenure-track academic positions How to secure tenure/promotion in the workplace Continuation of career training throughout a career Teach transferable skills
Mid-Career Scientists	Teaching/engaging in public policy Continue network development to build on research skills and promotions Train scientists to effectively communicate their science to non-scientists
Senior-Career Scientists/Emeritus	Create collaborations, especially with younger scientists who can benefit from advanced experience Be a mentor

Early-career scientists experience issues, such as cost-of-living pay increases, the need for more institutional support, and extramural funding opportunities, to guarantee successful lab research while early in the career, when publications are crucial to earning tenure and securing promotions. Mentors are an important aspect of early trainees, and the most effective mentors can guide academic professionals along the path to obtaining tenure. The need for career training should be continued over the length of a career, through the stages of career development from early career to mid-career to senior scientist.

Mid-career scientists who have participated in education, research, and publication for a longer period of time should play a greater role in communicating scientific discovery to the non-science public. They should also stay engaged in public policy to advocate for science, and they will continue to need mentors to maintain a network of peers with whom they can collaborate.

Senior-career scientists and retired scientists (emeritus) are a tremendous resource of mentorship to early scientists. Senior scientists have a wealth of experience to share with their younger peers, while early career scientists can share their newfound experiences in innovative microbiology research and technology. We should also keep in mind that even senior scientists can benefit from workshop training in the advancement of new technologies in the field.

## SESSION 3—TECHNOLOGICAL ADVANCEMENTS: HOW CAN THE MICROBIOLOGY PROFESSION BE TRAINED FOR RAPID TECHNOLOGICAL ADVANCEMENTS?

### Speaker highlights

Sandra Porter, Ph.D., president, Digital World Biology, LLC, and Shoreline Community College, shared her experiences in “Using Hackathons to Promote Learning and Catalyze Creativity.” Hackathons consist of teams that work together for a short period of time to create products, for example, crowdsourcing antibody engineering. She emphasized the importance of the roles of team leader, writer, data hunter, artist, and tech supporter as being necessary for a team’s success. Teams work best when each member has a specific role to accomplish and then reports their results back to the group.

Corine Jackman Burden, Ph.D., assistant professor, University of Maryland, Baltimore County, discussed “The Future-Proof Microbiologist: How to Stay Ahead in an Ever-Evolving Technological Landscape.” She described some of the new technologies that are impacting microbiology, including next-generation sequencing, bioinformatics, CRISPR-Cas9, and single-cell RNA sequencing. She recommended staying up to date in your field, using artificial intelligence (AI) if necessary, acquiring computational skills, embracing interdisciplinary collaborations, and continuing your education.

Noemí Kaoru Yokobori, Ph.D., associate researcher, Instituto Nacional de Enfermedades Infecciosas (INEI)-ANLIS and CONICET, Argentina, in her talk entitled “The Rising Tide of CRISPR-Cas in Tuberculosis Research” shared her experience using CRISPR-Cas9 in tuberculosis drug research to provide an example of how to train the next generation of microbiologists. She addressed the barriers to adopting new technologies and suggested ways to overcome these barriers. Ideas included attending conferences to be exposed to the new technologies on display, using NCBI and NIH webinars to help write more persuasive grant proposals, attending short courses to learn new methods, focusing on solving one problem at a time, seeking advice from someone who has already reached your goal, using generative AI to learn new things, and sharing expertise within your network or building a new network.

#### Small group discussion

Five virtual breakout rooms were available: Use of Artificial Intelligence in Education and Training, Current Barriers to Adopting New Technologies in Microbiology, Applying CRISPR to Research, Treatment and Diagnosis, and Keeping up with Innovations in Multi-omics. Each group addressed two prompt questions: what types of methods and formats of training are best for teaching these topics, and how can we integrate new technologies into our work?

### Key takeaways

Microbiology research is profoundly affected by AI. AI can be used as a resource to teach basic sciences to students, providing them with resources to learn more effectively. With advances in the collection of big data, AI is a useful tool to teach coding and other analytical skills. There will always be a need to teach the ethical use of generative AI so that critical thinking and problem solving are not replaced by AI tools (Table 3).

**TABLE 3:** Summary of technological advancements small group discussions

Topic	Suggestions
The Use of Artificial Intelligence	When is it appropriate/inappropriate to use AI in teaching and research? Need for critical thinking skills in using AI Need to use AI to teach microbiology content
Current Barriers to Adopting New Technologies	Develop a network of collaborators to teach new skills to those who are not prepared Incorporate exhibitor-led demonstrations and short courses on new technologies
Applying CRISPR	Accessibility of CRISPR tools at all workplaces and around the globe Need to teach the ethical use of genetic modification in research
Innovations in Multi-omics	Translation from the workbench to clinical medicine

The adoption of new and innovative technologies is critical to advancing our science. Private industries that produce new technologies can provide short courses and exhibitor-led demonstrations at microbiology research conferences to disseminate this information. As scientists embrace these new technologies, collaborations can be encouraged so as to establish a network of new trainees and advanced personnel.

CRISPR has been successful as a research tool at all levels. As new strategies evolve in the use of CRISPR, it is necessary to make these research advancements available to scientists around the world, regardless of the political economy. There will still be a need for open communication in the ethical use of CRISPR for genetic modifications.

The discussion on multi-omics included the areas of genomics, transcriptomics, proteomics, and metabolomics. The translation from lab bench to clinical practice is critical as we grow the basic science into applications in the prevention, diagnosis, and treatment of infectious diseases.

## SESSION 4—INTERDISCIPLINARY COLLABORATION: HOW CAN SCIENTISTS LEARN TO WORK IN GROWING INTERDISCIPLINARY COLLABORATIONS?

### Speaker highlights

Braden Tierney, Ph.D., executive director, Two Frontiers Project, shared “Organizational Strategies for Sustaining an Interdisciplinary Scientific Team.” He emphasized the need for overlapping skill sets, such as the combination of data science and DNA sequencing in research and development. He discussed the need for task management skills, division of labor, and the importance of having a complete understanding of the elements of a project and its timeline, as well as goal setting with mileposts along the way to map your progress. At the conclusion of a project, it is helpful to debrief and generate ideas for optimization for the next iteration.

Rodolphe Barrangou, Ph.D., professor, NC State University, discussed “The Many Hats Microbiologists Wear.” He focused on specific roles microbiologists play in their profession. These roles include researcher, teacher, ambassador, entrepreneur, coach, writer, administrator, and advocate. He recommended we ask ourselves what role we are currently filling, and which ones we need to develop next. He suggested setting deadlines and knowing what deliverables we should have to demonstrate mastery of before moving on to the next role.

Sandra Porter, Ph.D., president, Digital World Biology, LLC, and Shoreline Community College, discussed, in her talk entitled “Creative Ways to Connect,” how to improve establishing and building collaborations. She encouraged defining the goals and the scale of our project and the desired outcomes. She suggested using social media tools to find collaborators, and once the team is recruited, to divide the tasks and determine how to communicate.

#### Small group discussion

Two virtual breakout rooms were available: Building an Effective Academic Multi-disciplinary Team and Navigating the Intersection Between Academia with Local Government, Community, and Industry. Each group addressed two prompt questions: what are some professional/durable skills needed to facilitate different collaborations, and how are fruitful interdisciplinary collaborations established between stakeholders and integrated into current practices?

### Key takeaways

If we are to find solutions to the growing number of global problems, then an effective multi-disciplinary team between microbiologists and other academic disciplines is necessary. For meaningful collaborations to be established in academic and industry settings, an environment of trust between disciplines needs to be built. Computational and data literacy skills necessary to analyze big data sets can be provided in training seminars and shared between the stakeholders (Table 4).

**TABLE 4:** Summary of interdisciplinary collaborations small group discussions

Topic	Suggestions
Building an Effective Academic Multidisciplinary Team	Develop transferable skills useful in academia and industry Learn from each other in different disciplines Provide training in data literacy and computational methods
Navigating the Intersection among Academia and Local Government, Community, and Industry	How to prepare the non-scientist community to support science Develop transferable skills Define the many roles played by scientists in their communities Recruit industry onto ASM’s advisory boards and create programming for industry professionals at ASM Microbe Include interdisciplinary research as keynote/plenary sessions for ASM Microbe

In addition, we should encourage more collaborations between academia, local government, citizen science, and industry. Outreach to all stakeholders will be necessary to teach public citizens the need for greater scientific research and the benefit to the community as a whole. These grassroots efforts can start in local communities where scientists define the many roles they play. This, in turn, will encourage non-scientists to promote the need for more scientific research. The ASM annual meeting can become a venue for academic research and industry-led research to come together and share their findings.

## DEMOGRAPHIC COMPOSITION OF RETREAT ATTENDEES

**Fig 1:**
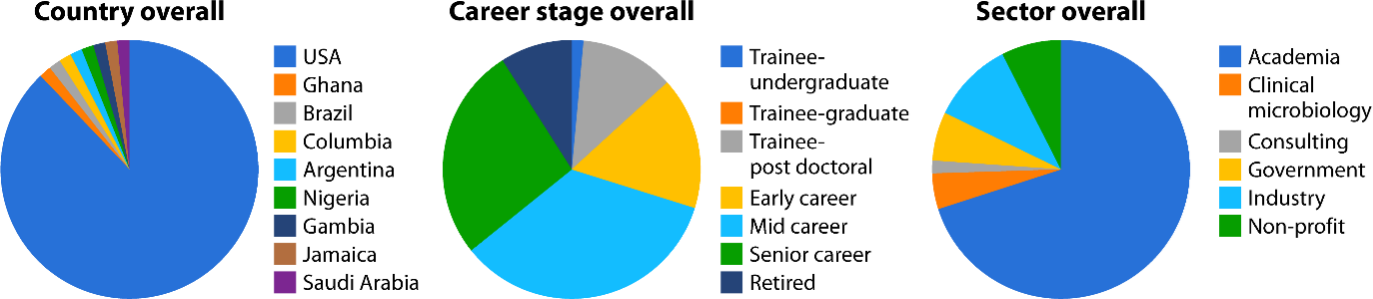
Demographics of the POM retreat participants.

Most of the attendees were from the United States (88%), with other countries contributing in total to 4.5% (Fig. 1). This distribution did not vary substantially throughout the four sessions (Fig. S1).

In terms of the career stage, more than half of the participants were in the mid- and senior-career stage, followed by early-career. More than half of the attendees of Session 3 on Technological Advancements were mid-career professionals. The greatest number of trainees (postdoctoral level) attended during Session 2, which focused on professional development. Participants belonging to the academic sector dominated the demographics at almost 75%.

## FEEDBACK FROM THE ASM COMMUNITY

A wrap-up session was held in person at the 2025 ASM Microbe Meeting in Los Angeles, during which highlights from the POM retreat were shared with attendees and they were asked for their input. Below is a summary of the feedback from the participants at that session.

### Workforce development

#### Jobs in academia

There are still many good jobs in academia, including jobs outside of the United States. We should speak optimistically about these positions, especially to trainees who are exploring these opportunities. The positive aspects that academic positions offer include a good work-life balance and capturing the joy of being a microbiologist.

There are non-microbiologists (for example, engineers and food scientists) who work with microbiologists. We need to ensure that non-microbiologists are trained properly to do microbiology work.

#### Jobs in government

The largest governmental department is the Department of Defense. There are microbiologists in the Army and Navy who are distinct from those who work in the Department of Health and Human Services. We need to be better at communicating these job opportunities to trainees.

#### Jobs in industry

Some of the selling points for careers in industry include mobility, attractive salaries, and more opportunities for professional development and promotion. We can help trainees identify the skills they currently have and direct them toward jobs that best utilize these skills. Internships for industry jobs could be provided, during which broad skills could be taught. Multi-year internships, allowing students to return to the same workplace and offering competitive pay, would be attractive. Advanced internships in which a student stays for 6 months at a job could also be considered. Pipelines into industry through universities could be better developed, as well as including more industry representation at university career fairs. By integrating the availability of industry jobs into the undergraduate curriculum, one can prompt students to think about careers outside of academia early enough in their careers to start preparing.

Industry-focused tracks at ASM Microbe could be created. Industry does not send many attendees to ASM Microbe because it does not seem to be a community for them, and there are not enough sessions devoted to their interests. Ideas for industry-related sessions that could benefit trainees and early-career industry individuals could include preservation, biosafety, microbial risk, Biopharma, AgBiotech, and environmental systems.

### Professional and career skills development

Career development can start as early as secondary education in the United States. We need to teach early trainees about careers in microbiology. Career fairs can target community college audiences, as well as undergraduates, at 4-year institutions. ASM should consider doing more for early STEM adoptees in K-12 and their teachers.

Having an online resource portal of workshops that only ASM members can log into is an idea. Resources could be available to non-ASM members through open education platforms. These could also be available to K-12 and undergraduate audiences. Outreach to K-12 through university career fairs could provide them with information about jobs in microbiology. An effort to retain STEM students at community colleges (via CUREs) should be implemented.

It will become more important in the future to prepare for jobs that do not yet exist. Because we do not yet know what those jobs will be, we may feel limited in how we prepare for them. We do know, though, that those jobs will require many of the transferable skills that we can prepare for now. Therefore, the skills to master these jobs may be taught now. We should investigate what these skills are and how to develop them today. Short courses, webinars, and online training to learn these skills could be developed outside of the workplace and the traditional classroom. Regional Branch meetings would be a good place to introduce these workshops since we should be targeting trainees and early-career scientists, and they generally attend Branch meetings more often than ASM Microbe.

Among the many functions performed by microbiologists, we should mention researcher, teacher, writer, ambassador, entrepreneur, coach, administrator, and advocate. Developing skills in these areas now is more important than ever because employees are switching careers from academia to industry and back again from industry to academia. Although industry skills are often the same as academia (for example, budget management, time management, working toward a deadline, oral/written communication skills, teamwork, and employee management), one main difference is that industry seeks upfront recommendations and result projections on projects. Communication to industry stakeholders is also different.

### Technological advancements

In an era defined by rapid technological advancement and information abundance, the question of how we learn has become more critical than ever. Central to this inquiry is the concept of metacognition—the ability to reflect on and regulate one’s own learning processes. As students and professionals alike navigate increasingly complex scientific landscapes, fostering metacognitive skills is essential for lifelong learning and adaptability. Included in this lifelong learning is a recurring challenge in STEM education: students’ difficulty in interpreting scientific figures and data visualizations. Journals and professional societies can play a pivotal role by offering guidance and resources, such as figure interpretation guides or annotated examples, to demystify complex visuals and enhance data literacy.

To extend these benefits beyond flagship events, regional Branch meetings could incorporate more exhibitor-led demonstrations and short courses, offering trainees and early-career scientists exposure to essential tools and techniques. Complementing these in-person experiences with online training modules can ensure broader accessibility and continuity of learning.

The future of AI in research shows promise, but we are aware of issues concerning data privacy, algorithmic bias, and the erosion of critical thinking if relied upon excessively. As educators, mentors, and supervisors, it is our responsibility to address questions such as how we responsibly use AI and when is it appropriate for students to use AI in their education? In sum, a multifaceted approach combining metacognition, ethical AI integration, and experiential learning will enhance student learning as they apply innovation in the life sciences.

### Interdisciplinary collaboration

The evolving landscape of microbiology and the broader life sciences demands a workforce equipped with transferable skills in leadership, analytical skills, and communication. Although data collection has become increasingly accessible, there remains a critical shortage of professionals capable of interpreting complex data sets. Addressing this gap requires targeted training in data literacy and computational methods, beginning at the undergraduate level and extending through later career stages.

To bridge the divide between academic training and real-world applications, stronger collaborations between academia and industry are essential. Undergraduate students will benefit from experiences in clinical laboratories and industry-led workshops. However, the cultivation of scientific curiosity and career awareness must begin even earlier. K-12 outreach initiatives that introduce students to microbial science and its societal relevance can inspire future generations of microbiologists.

Scientific conferences such as ASM Microbe serve as vital platforms for interdisciplinary exchange and professional growth. Inviting non-microbiologists to deliver keynote addresses can expose attendees to novel perspectives and foster cross-disciplinary innovation. Similarly, plenary sessions that highlight collaborative research across ASM units can demonstrate the power of integrated approaches to complex scientific challenges.

Solving global problems, from antimicrobial resistance to climate change, requires multidisciplinary teams that include not only scientists but also community stakeholders. Effective science communication is therefore paramount. Microbiologists must learn to convey their science in accessible language, while also listening to the needs and insights of non-science experts. This reciprocal exchange can be facilitated by providing community collaborators with foundational microbiological vocabulary and context, enabling them to participate meaningfully in scientific discourse.

Fostering a culture of interdisciplinary collaboration, grounded in strong technical skills and mutual respect, will be essential for advancing microbial science and addressing the pressing challenges of our time. The ability to build a network, establish collaborations to gain new skills, and apply them to your research is now an important skill at all career stages. Sometimes, a barrier to developing a new research program is not just funding but having the skills necessary to compete for funding. Networking can provide a resource for skills that will enhance any research discovery program.

## DISCUSSION AND SCIENTIFIC TRENDS

### Setting the stage for the future of the POM

The future of microbiology depends on cultivating a workforce that is not only technically skilled but also engaged, adaptable, and purpose-driven.

In 2021, microbiologists were generally satisfied with their work (2). Of those microbiologists who were not satisfied in 2019, every dissatisfied microbiologist noted a lack of opportunities for advancement as the cause of their dissatisfaction, followed by 78% citing the lack of intellectual challenges.

Satisfaction in the microbiology workforce can be enhanced through (i) clear career pathways that span from academia, industry, clinical microbiology, public health, to policy and advocacy, (ii) mentorship and leadership training, especially for early-career scientists, (iii) flexible work environments that support work-life balance and mental well-being, and (iv) cross-sector mobility, allowing professionals to transition between research, regulatory, management, and applied roles.

ASM and other institutions increasingly recognize the need to connect microbiologists across sectors to address global challenges, such as antimicrobial resistance and emerging infectious diseases.

Technological advancements call for microbial scientists to be prepared for rapid innovation. To keep pace, microbiologists must engage in continuous learning, supported by modular training, certifications, and hands-on workshops. Microbiology is being transformed by technological breakthroughs, including

- High-throughput sequencing and bioinformatics, which require training in data science and computational biology.
- CRISPR and gene editing, enabling precise genetic manipulation for health, agriculture, and environmental applications.
- Synthetic biology and systems biology, which blend engineering with microbiology to design novel biological systems, and
- AI and machine learning, which are revolutionizing diagnostics, microbial ecology, and drug discovery.

The excitement due to the potential for AI to improve efficiency and quality in the clinical microbiology practice was recognized by the participants of the ASM Clinical Microbiology Open 2024. They recognized that AI is beginning to change the practice of clinical microbiology, including quality monitoring, information retrieval and communication, and interpretation of visual data. Furthermore, current and anticipated challenges regarding AI use in clinical practice include the lack of local laboratory expertise in AI development, limited experience in maintaining and adapting algorithms as data evolve over time, and an uncertain regulatory environment (11). As technological advancements and automation would be taking care of operative tasks, the future of the microbiologist in medicine would be cardinal in providing safety and responsible information (12).

The integration of AI into microbiology has the potential to transform and advance our understanding and treatment of microbial systems, marking a pivotal moment in the evolution of the entire field, including research, diagnostics, and public health. AI technologies are already being applied to a wide range of microbiological challenges, including predicting drug targets and vaccine candidates, identifying pathogens responsible for infectious diseases, classifying antimicrobial resistance, forecasting disease outbreaks, analyzing microbial interactions, and ensuring quality control and regulatory compliance. These advances promise to enhance our ability to detect, understand, and treat microbial systems with unprecedented precision. However, the integration of AI is not without challenges. Issues, such as data heterogeneity, lack of model transparency, and ethical concerns, must be addressed to ensure responsible and effective use. Success in this domain will require interdisciplinary collaboration, robust data governance, and rigorous validation of AI models (13, 14).

It is interesting to witness the existing concerns about the mobility from academia to industry and vice versa in the microbiology profession. Scientists transitioning from academia to industry may need to reframe their academic experience in terms of teamwork, collaboration, and project management skills highly valued in corporate environments (15). Industry roles often prioritize deliverables and deadlines over publications. Although many microbiology doctoral graduates are interested in industry, few receive adequate career guidance. Early preparation and networking with industry professionals should be key to a successful transition, so they can leverage academic training, such as data analysis, grant writing, and team leadership, for entrepreneurial and industry roles (16).

Professional development programs should also address non-traditional career paths, including roles in regulatory affairs and policy, science writing, and patent law. Solving today’s complex problems requires collaborative teams that span disciplines and sectors. Microbiologists must work alongside engineers, data scientists, and chemists to develop new tools and technologies; public health officials and clinicians to translate research into practice; and educators and communicators to engage the public and policymakers.

Effective collaboration also demands shared language and mutual respect. Training in science communication, cultural competency, and stakeholder engagement is essential to ensure that microbiologists can both contribute to and learn from diverse perspectives. The importance of communication, adaptability, and vision in both sectors is a topic that was mentioned in the previous POM retreat report (10).

## KEY RECOMMENDATIONS FROM POM TO ASM

Professional conferences like ASM Microbe present unique opportunities to bridge the gap between learning and application. To support the evolving needs of the POM, ASM can enhance its annual meeting by offering targeted programming, including networking opportunities, and expanding educational resources. These efforts would increase professional engagement, foster interdisciplinary collaboration, and broaden access to microbiological knowledge and tools.

A key area of growth lies in the development of industry-focused tracks tailored to non-academic professionals. Session topics, such as microbial preservation, biosafety, risk assessment, and industrial microbiology, can provide relevant content for professionals in applied areas, encouraging broader participation from sectors beyond academia.

Meanwhile, the Exhibit Fair and Industry Lounge and Learn sessions can be reimagined as hubs for bite-sized, hands-on demonstrations of cutting-edge technologies, such as nanopore sequencing, single-cell analytics, and mass spectrometry. These experiences can spark curiosity and provide tangible entry points into new areas of research. At the same time, these group formats could reduce the pressure of one-on-one interactions and allow attendees to explore new tools in a low-commitment, high-impact setting.

The formation of Special Interest Groups can foster peer networks and sustained engagement around emerging topics. These could be organized around thematic or technical areas, enabling attendees to build networks, share resources, and initiate collaborations. Similarly, multidisciplinary plenary sessions that span multiple ASM units can showcase how diverse scientific approaches converge to solve complex problems, inspiring attendees to think beyond disciplinary boundaries.

To inspire and inform, ASM can also spotlight success stories of individuals who leveraged new skills or technologies gained at ASM Microbe to advance their careers or research. These narratives can serve as powerful motivators and illustrate the tangible impact of professional development.

The role of educators, from K-12 to graduate levels, should be more fully integrated into the ASM Microbe meeting. Although ASMCUE serves as a dedicated space for undergraduate education, ASM Microbe can offer complementary programming that highlights pedagogical innovation, curriculum development, and resource sharing. A Microbe Library or Resource Portal, accessible to ASM members, would be especially valuable for under-resourced educators at community colleges and K-12 institutions.

## CONCLUSION

The ultimate goals of these initiatives are to increase networking, grow membership, expand exposure to career opportunities, and integrate microbiology more deeply into interdisciplinary research. By aligning its annual meeting with the diverse needs of its community, ASM can continue to lead in shaping the future of the microbial sciences, fulfilling its ultimate goal of benefiting humankind through bridging science and society. All of which will better equip us to recognize and solve the global challenges of our time.

## SUPPLEMENTAL MATERIAL

Figure S1 msphere.00654-25-s0001.docx Demographics of the POM retreat participants.

ASM does not own the copyrights to Supplemental Material that may be linked to, or accessed through, an article. The authors have granted ASM a non-exclusive, world-wide license to publish the Supplemental Material files. Please contact the corresponding author directly for reuse.
