# Supplementary material for: Perspectives on the future of the profession of microbiology
Source: mSphere. 2025 Nov 25;10(12):e00654-25. doi: 10.1128/msphere.00654-25 (PMC12724239; doi:10.1128/msphere.00654-25)
Supplement: Figure S1 — Demographics of the POM retreat participants. [file msphere.00654-25-s0001.docx]

Suppl. Figure 1.


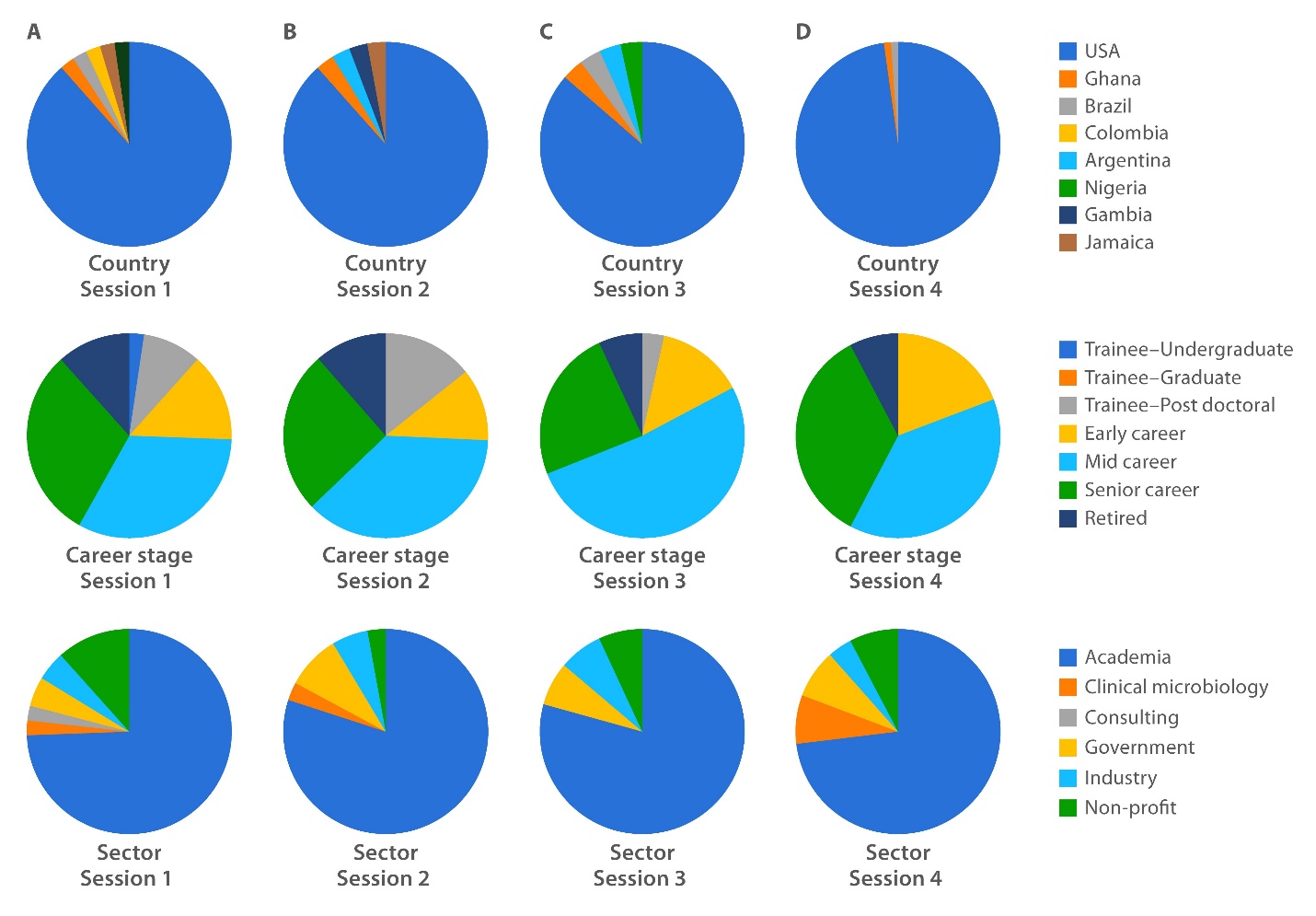


Suppl. Figure 1. Demographics of the POM retreat participants during the first (A), second (B), third (C), and fourth (D) sessions.
